# Storage Capacities of Twin-Multistate Quaternion Hopfield Neural Networks

**DOI:** 10.1155/2018/1275290

**Published:** 2018-11-01

**Authors:** Masaki Kobayashi

**Affiliations:** Mathematical Science Center, University of Yamanashi, Takeda 4-3-11, Kofu, Yamanashi 400-8511, Japan

## Abstract

A twin-multistate quaternion Hopfield neural network (TMQHNN) is a multistate Hopfield model and can store multilevel information, such as image data. Storage capacity is an important problem of Hopfield neural networks. Jankowski et al. approximated the crosstalk terms of complex-valued Hopfield neural networks (CHNNs) by the 2-dimensional normal distributions and evaluated their storage capacities. In this work, we evaluate the storage capacities of TMQHNNs based on their idea.

## 1. Introduction

A complex-valued Hopfield neural network (CHNN) is a multistate model of Hopfield neural network. CHNNs can deal with multilevel information and have been applied to the storage of image data [1–7]. They have also been extended using Clifford algebra, which includes a complex field, hyperbolic algebra, and quaternion field. Several models of hyperbolic Hopfield neural network have been proposed [8–11]. Isokawa et al. [12] proposed quaternion Hopfield neural networks (QHNNs) employing the split activation function. Minemoto et al. [13] studied QHNNs using the polar-represented activation function. Several other models of QHNNs have also been proposed [14–17]. In this work, we study the twin-multistate quaternion Hopfield neural networks (TMQHNNs) [18]. The neuron of a TMQHNN consists of a pair of complex-valued multistate neurons. The TMQHNN requires only half the connection weight parameters of the CHNN.

Storage capacity is an important issue in Hopfield neural networks. When a Hopfield neural network is given a training pattern, the weighted sum input is decomposed into main and crosstalk terms. The main term enables the Hopfield neural network to memorize the training patterns. The crosstalk term interferes with the storage of training patterns. The storage capacity of conventional Hopfield neural network has been investigated by evaluating the crosstalk term [19]. Jankowski et al. [1] and Kobayashi [20] applied this technique to CHNNs and rotor Hopfield neural networks (RHNNs), respectively. The RHNN is an extension of the CHNN using vectors and matrices [21]. In this work, we provide the Hebbian learning rule for TMQHNNs and evaluate the storage capacity based on Jankowski's concept. In the case of TMQHNNs, the cross talk term is decomposed into two complex parts. By evaluating both parts, we determine the storage capacity of TMQHNNs. The theory suggests that TMQHNNs have half the storage capacities of CHNNs. In addition, we compared the storage capacities of CHNNs and TMQHNNs by computer simulation.

The rest of this paper is organized as follows: Sections 2 and 3 introduce CHNNs and TMQHNNs, respectively.  Section 4 provides the Hebbian learning rule for the TMQHNN and evaluates the storage capacity. It also contains descriptions of the computer simulations conducted to verify our analysis.  Section 5 concludes this paper.

## 2. Complex-Valued Hopfield Neural Networks

The CHNNs are briefly described [1]. Let *z*_*a*_ and *w*_*ab*_ be the state of neuron *a* and the connection weight from neuron *b* to neuron *a*, respectively. The weighted sum input to neuron *a* is given by

(1) $$\begin{array}{c} I_{a}=\overset{N_{\text{C}}}{\underset{b=1}{\sum }}w_{ab}z_{b}, \end{array}$$

where *N*_C_ is the number of neurons. For the resolution factor *K*, we define *θ*_*K*_=*π*/*K*. For the weighted sum, input *I*=*r*exp(*iθ*), where *r* ≥ 0 and 0 ≤ *θ* < 2*π*; the complex-valued multistate activation function is defined by

(2) $$\begin{array}{c} f_{\text{C}}\left(I\right)=\left\{\begin{array}{cc} 1 & 0\leq \theta <\theta_{\text{K}},\\ \exp\left(2i\theta_{\text{K}}\right) & \theta_{\text{K}}\leq \theta <3\theta_{\text{K}},\\ \vdots & \vdots \\ \exp\left(2\left(K-1\right)i\theta_{\text{K}}\right) & \left(2K-3\right)\theta_{\text{K}}\leq \theta <\left(2K-1\right)\theta_{\text{K}},\\ 1 & \left(2K-1\right)\theta_{\text{K}}\leq \theta <2\pi . \end{array}\right. \end{array}$$

We define the set of neuron states and denote it as follows: *S*={1, exp(2*iθ*_K_),…, exp(2(*K* − 1)*iθ*_K_)}. The connection weights must satisfy the following conditions:

(3) $$\begin{array}{c} \bar{w_{ab}}=w_{ba}, \end{array}$$

(4) $$\begin{array}{c} w_{aa}\geq 0. \end{array}$$

Then, the CHNN converges to a fixed point.

Let **z**^*p*^=(*z*_1_^*p*^, *z*_2_^*p*^,…, *z*_*N*_C__^*p*^)(1 ≤ *p* ≤ *P*_C_) be the *p*th training pattern, where *P*_C_ is the number of training patterns. The Hebbian learning rule is defined as

(5) $$\begin{array}{c} w_{ab}=\frac{1}{N_{\text{C}}}\overset{P_{\text{C}}}{\underset{p=1}{\sum }}z_{a}^{p}\bar{z_{b}^{p}}. \end{array}$$

Then, the connection weights satisfy $\bar{w_{ab}}=w_{ba}$. Giving the *q*th training pattern to the CHNN, the weighted sum input to neuron *a* is

(6) $$\begin{array}{c} I_{a}^{q}=\overset{N_{\text{C}}}{\underset{b=1}{\sum }}w_{ab}z_{b}^{q} \end{array}$$

(7) $$\begin{array}{c} =z_{a}^{q}+\frac{1}{N_{\text{C}}}\underset{p\neq q}{\sum }\overset{N_{\text{C}}}{\underset{b=1}{\sum }}z_{a}^{p}\bar{z_{b}^{p}}z_{b}^{q}. \end{array}$$

The second term of (7) is referred to as the crosstalk term. The crosstalk term interferes with the storage of training patterns. We define

(8) $$\begin{array}{c} A_{a}^{q}=\frac{1}{N_{\text{C}}}\underset{p\neq q}{\sum }\overset{N_{\text{C}}}{\underset{b=1}{\sum }}z_{a}^{p}\bar{z_{b}^{p}}\bar{z_{a}^{q}}z_{b}^{q}. \end{array}$$

Then, we have

(9) $$\begin{array}{c} I_{a}^{q}=z_{a}^{q}\left(1+A_{a}^{q}\right). \end{array}$$

If |arg(1+*A*_*a*_^*q*^)| < *θ*_K_, then we have *f*_C_(*I*_*a*_^*q*^)=*z*_*a*_^*q*^. Therefore, if |arg(1+*A*_*a*_^*q*^)| < *θ*_K_ for all *a*, the *q*th training pattern is a fixed point. We regard *N*_C_*A*_*a*_^*q*^ as the summation of *P*_C_*N*_C_ random variables of *V* for simplicity, although the summation consists of exactly (*P* − 1)*N*_C_ terms. The real and imaginary parts of each random variable have the equal variance *σ* and do not have correlations. Setting *P*_C_=*αN*_C_, *N*_C_*A*_*a*_^*q*^ is regarded as the summation of *αN*_C_^2^ random variables. Then, we have

(10) $$\begin{array}{c} \frac{1}{\sqrt{\alpha }}A_{a}^{q}=\frac{1}{\sqrt{\alpha }N_{\text{C}}}\underset{p\neq q}{\sum }\overset{N_{\text{C}}}{\underset{b=1}{\sum }}z_{a}^{p}\bar{z_{b}^{p}}\bar{z_{a}^{q}}z_{b}^{q}. \end{array}$$

Let *X*_*A*_ and *Y*_*A*_ be the real and imaginary parts of $\left(1/\sqrt{\alpha }\right)A_{a}^{q}$, respectively. From the central limit theorem, we have

(11) $$\begin{array}{c} \left(X_{A},Y_{A}\right)∼\frac{1}{2\pi \sigma^{2}}\exp\left(-\frac{X_{A}^{2}+Y_{A}^{2}}{2\sigma^{2}}\right). \end{array}$$

For *A*_*a*_^*q*^=*X*_*A*_′+*Y*_*A*_′*i*, we have

(12) $$\begin{array}{c} \left(X_{A}^{\prime },Y_{A}^{\prime }\right)∼\frac{1}{2\alpha \pi \sigma^{2}}\exp\left(-\frac{{X_{A}^{\prime }}^{2}+{Y_{A}^{\prime }}^{2}}{2\alpha \sigma^{2}}\right). \end{array}$$

## 3. Twin-Multistate Quaternion Hopfield Neural Networks

A quaternion is expressed by *q*=*q*_0_+*q*_1_*i*+*q*_2_*j*+*q*_3_*k* using real numbers *q*_0_, *q*_1_, *q*_2_, and *q*_3_. The imaginary units *i*, *j*, and *k* satisfy the following properties:

(13) $$\begin{array}{c} i^{2}=j^{2}=k^{2}=-1,\\ ij=-ji=k,\\ jk=-kj=i,\\ ki=-ik=j. \end{array}$$

The quaternions satisfy the associative and distributive laws. For a complex number *c*, we have the important equality:

(14) $$\begin{array}{c} jc=\bar{c}j. \end{array}$$

Putting *x*=*q*_0_+*q*_1_*i* and *y*=*q*_2_+*q*_3_*i*, the quaternion *q* is described as *q*=*x*+*yj*. For quaternions *x*+*yj* and *x*′+*y*′*j*, the addition and multiplication are described as

(15) $$\begin{array}{c} \left(x+yj\right)+\left(x^{\prime }+y^{\prime }j\right)=\left(x+x^{\prime }\right)+\left(y+y^{\prime }\right)j,\\ \left(x+yj\right)\left(x^{\prime }+y^{\prime }j\right)=\left(xx^{\prime }-y\bar{y^{\prime }}\right)+\left(xy^{\prime }+\bar{x^{\prime }}y\right)j. \end{array}$$

The conjugate of *q* is defined as

(16) $$\begin{array}{c} \bar{q}=q_{0}-q_{1}i-q_{2}j-q_{3}k\\ =\bar{x}-yj. \end{array}$$

Then, we have the equality

(17) $$\begin{array}{c} q\bar{q}=x\bar{x}+y\bar{y}\\ ={\left|x\right|}^{2}+{\left|y\right|}^{2}. \end{array}$$

In the TMQHNNs, the neuron states and connection weights are represented by quaternions. These neuron states and connection weights are denoted in the same way as those of CHNNs. The number of neurons in a TMQHNN is denoted as *N*_Q_. The weighted sum input to neuron *a* is given by

(18) $$\begin{array}{c} I_{a}=\overset{N_{\text{Q}}}{\underset{b=1}{\sum }}w_{ab}z_{b}. \end{array}$$

For the weighted sum, input *I*=*I*_*x*_+*I*_*y*_*j*, the activation function is defined as

(19) $$\begin{array}{c} f_{\text{Q}}\left(I\right)=f_{\text{C}}\left(I_{x}\right)+f_{\text{C}}\left(I_{y}\right)j. \end{array}$$

Therefore, the set of neuron states is *S*+*Sj*. The connection weights must satisfy conditions (3) and (4). Then, the TMQHNN converges to a fixed point.

## 4. Storage Capacity of Twin-Multistate Quaternion Hopfield Neural Networks

We provide the Hebbian learning rule for TMQHNNs. Let **z**^*p*^=(*z*_1_^*p*^, *z*_2_^*p*^,…, *z*_*N*_Q__^*p*^)(1 ≤ *p* ≤ *P*_Q_) be the *p*th training pattern, where *P*_Q_ is the number of training patterns. The Hebbian learning rule is given by

(20) $$\begin{array}{c} w_{ab}=\frac{1}{2N_{\text{Q}}}\overset{P_{\text{Q}}}{\underset{p=1}{\sum }}z_{a}^{p}\bar{z_{b}^{p}}. \end{array}$$

Then, the connection weights satisfy ${\bar{w}}_{ab}=w_{ba}$. Giving the *q*th training pattern to the TMQHNN, the weighted sum input to neuron *a* is

(21) $$\begin{array}{c} I_{a}^{q}=\underset{b\neq a}{\sum }w_{ab}z_{b}^{q} \end{array}$$

(22) $$\begin{array}{c} =z_{a}^{q}+\frac{1}{2N_{\text{Q}}}\underset{p\neq q}{\sum }\overset{N_{\text{Q}}}{\underset{b=1}{\sum }}z_{a}^{p}\bar{z_{b}^{p}}z_{b}^{q}. \end{array}$$

The second term of (22) is also referred to as the crosstalk term and interferes the storage of training patterns. We decompose the quaternion *z*_*a*_^*p*^ into a pair of complex numbers by *z*_*a*_^*p*^=*x*_*a*_^*p*^+*y*_*a*_^*p*^*j* to investigate the storage capacity. Then, we have

(23) $$\begin{array}{c} I_{a}^{q}=x_{a}^{q}+y_{a}^{q}j+\frac{1}{2N_{\text{Q}}}\underset{p\neq q}{\sum }\overset{N_{\text{Q}}}{\underset{b=1}{\sum }}\left(x_{a}^{p}+y_{a}^{p}j\right)\left(x_{b}^{p}-y_{b}^{p}j\right)\left(x_{b}^{q}+y_{b}^{q}j\right)\\ =x_{a}^{q}+\frac{1}{2N_{\text{Q}}}\underset{p\neq q}{\sum }\overset{N_{\text{Q}}}{\underset{b=1}{\sum }}\left(x_{a}^{p}x_{b}^{p}x_{b}^{q}+x_{a}^{p}y_{b}^{p}\bar{y_{b}^{q}}-y_{a}^{p}\bar{x_{b}^{p}}\bar{y_{b}^{q}}+y_{a}^{p}\bar{y_{b}^{p}}x_{b}^{q}\right)+y_{a}^{q}j+\frac{1}{2N_{\text{Q}}}\underset{p\neq q}{\sum }\overset{N_{\text{Q}}}{\underset{b=1}{\sum }}\left(x_{a}^{p}x_{b}^{p}y_{b}^{q}-x_{a}^{p}y_{b}^{p}\bar{x_{b}^{q}}-y_{a}^{p}\bar{x_{b}^{p}}\bar{x_{b}^{q}}+y_{a}^{p}\bar{y_{b}^{p}}y_{b}^{q}\right)j. \end{array}$$

We define

(24) $$\begin{array}{c} B_{a}^{q}=\frac{1}{2N_{\text{Q}}}\underset{p\neq q}{\sum }\overset{N_{\text{Q}}}{\underset{b=1}{\sum }}\left(x_{a}^{p}x_{b}^{p}x_{b}^{q}+x_{a}^{p}y_{b}^{p}\bar{y_{b}^{q}}-y_{a}^{p}\bar{x_{b}^{p}}\bar{y_{b}^{q}}+y_{a}^{p}\bar{y_{b}^{p}}x_{b}^{q}\right)\bar{x_{a}^{q}},\\ C_{a}^{q}=\frac{1}{2N_{\text{Q}}}\underset{p\neq q}{\sum }\overset{N_{\text{Q}}}{\underset{b=1}{\sum }}\left(x_{a}^{p}x_{b}^{p}y_{b}^{q}-x_{a}^{p}y_{b}^{p}\bar{x_{b}^{q}}-y_{a}^{p}\bar{x_{b}^{p}}\bar{x_{b}^{q}}+y_{a}^{p}\bar{y_{b}^{p}}y_{b}^{q}\right)\bar{y_{a}^{q}}. \end{array}$$

Then, we have

(25) $$\begin{array}{c} I_{a}^{q}=x_{a}^{q}\left(1+B_{a}^{q}\right)+y_{a}^{q}\left(1+C_{a}^{q}\right)j. \end{array}$$

If |arg(1+*B*_*a*_^*q*^)| < *θ*_K_ and |arg(1+*C*_*a*_^*q*^)| < *θ*_K_, then we have *f*_Q_(*I*_*a*_^*q*^)=*z*_*a*_^*q*^. We regard 2*N*_Q_*B*_*a*_^*q*^ and 2*N*_Q_*C*_*a*_^*q*^ as the summations of 4*N*_Q_*P*_Q_ random variables of *S*. Then, *B*_*a*_^*q*^ and *C*_*a*_^*q*^ follow the same distributions. Thus, we can discuss only *B*_*a*_^*q*^. If the TMQHNN is used instead of the CHNN, *N*_C_=2*N*_Q_ is required, since a twin-multistate quaternion neuron consists of two complex-valued multistate neurons. Setting *P*_Q_=*βN*_C_=2*βN*_Q_, 2*N*_Q_*B*_*a*_^*q*^ is regarded as the summation of 8*βN*_Q_^2^ random variables, and we have

(26) $$\begin{array}{c} \frac{1}{\sqrt{2\beta }}B_{a}^{q}=\frac{1}{2\sqrt{2\beta }N_{\text{Q}}}\underset{p\neq q}{\sum }\overset{N_{\text{Q}}}{\underset{b=1}{\sum }}\left(x_{a}^{p}x_{b}^{p}x_{b}^{q}+x_{a}^{p}y_{b}^{p}\bar{y_{b}^{q}}-y_{a}^{p}\bar{x_{b}^{p}}\bar{y_{b}^{q}}+y_{a}^{p}\bar{y_{b}^{p}}x_{b}^{q}\right)\bar{x_{a}^{q}}. \end{array}$$

Let *X*_*B*_ and *Y*_*B*_ be the real and imaginary parts of $\left(1/2\sqrt{\beta }\right)B_{a}^{q}$, respectively. From the central limit theorem, we have

(27) $$\begin{array}{c} \left(X_{B},Y_{B}\right)∼\frac{1}{2\pi \sigma^{2}}\exp\left(-\frac{X_{B}^{2}+Y_{B}^{2}}{2\sigma^{2}}\right). \end{array}$$

Putting *B*_*a*_^*q*^=*X*_*B*_′+*Y*_*B*_′*i*, we have

(28) $$\begin{array}{c} \left(X_{B}^{\prime },Y_{B}^{\prime }\right)∼\frac{1}{4\beta \pi \sigma^{2}}\exp\left(-\frac{X_{B}^{\prime 2}+Y_{B}^{\prime 2}}{4\beta \sigma^{2}}\right). \end{array}$$

We require the same distributions for (12) and (22) and obtained *α*=2*β*. Thus, the CHNN has double the storage capacity of the TMQHNN.

**Figure 1:**
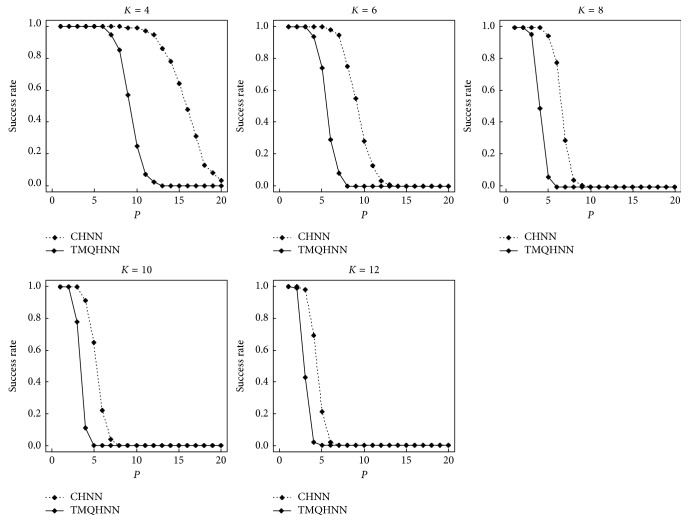
The simulation result for storage capacities.

Computer simulations were conducted to verify our analysis. *K* was varied from 4 − 12 in steps of 2, and *P* was varied from 1 − 20. For each *K* and *P*, 100 sets of training patterns were generated randomly; the number of trials was 100. The CHNN and TMQHNN attempted to store the training patterns by the Hebbian learning rule. If all the training patterns were fixed, the trial was regarded as successful, otherwise, as failed.  Figure 1 shows the simulation results. The horizontal and vertical axes indicate the number of training patterns and success rate, respectively. The simulation results showed that the storage capacity of the TMQHNN was a bit larger than half that of the CHNN.

## 5. Conclusions

A TMQHNN needs only half the connection weight parameters of CHNN and is expected to have a smaller storage capacity. However, the storage capacity had not yet been analyzed. In this work, we defined the Hebbian learning rule for TMQHNNs and analyzed their storage capacity. The analysis demonstrated that a TMQHNN had half of the storage capacity of a CHNN. In addition, a computer simulation was conducted to verify our analysis. The simulation results confirmed our analysis. In future, we intend to study the storage capacity using different methods [19, 22, 23].

## Data Availability

No data were used to support this study.
